# Aspiration thrombectomy versus stent retriever thrombectomy as a first-line approach for cardiogenic cerebral embolism and cryptogenic stroke in large vessels of the anterior circulation

**DOI:** 10.3389/fneur.2023.1324725

**Published:** 2024-01-15

**Authors:** Peng Zhiqiang, Chen Junchen, Cao Wenying, Zhao Dongqing, Ma Mi, Li Qiaowei, Zhu Zhenzhen, He Wanyi, Yang Biqing, Cao Minqi

**Affiliations:** ^1^Department of Stroke Center, Guangzhou Panyu Central Hospital, Guangzhou, Guangdong, China; ^2^Department of Neurosurgery, Zhongshan Hospital of Traditional Chinese Medicine, Zhongshan, Guangdong, China; ^3^Department of Intensive Care Unit, The Second Affiliated Hospital of Guangzhou Medical University, Guangzhou, Guangdong, China

**Keywords:** aspiration thrombectomy, stent retriever thrombectomy, acute large vessel occlusion stroke, anterior circulation, cardiogenic cerebral embolism, cryptogenic stroke

## Abstract

**Subject:**

This study aims to compare the clinical efficacy of aspiration thrombectomy and stent retriever thrombectomy as first-line approaches for anterior circulation large vessel cardiogenic cerebral embolism and cryptogenic stroke.

**Method:**

This retrospective observational study included patients with anterior circulation large vessel cardiogenic cerebral embolism and cryptogenic stroke treated with endovascular therapy. Patients were grouped according to the first-line approach they received: aspiration thrombectomy or stent retriever thrombectomy. The primary outcome measure was the change in the National Institute of Health Stroke Scale (NIHSS) score from preoperative to immediate postoperative and from preoperative to discharge. Secondary indicators included the rate of favorable prognosis at discharge [Modified Rankin Scale (mRS) score ≤ 2], successful vessel recanalization rate [modified Treatment in Cerebral Ischemia (mTICI) score ≥ 2b], time from successful femoral artery puncture to successful vessel recanalization, and perioperative complications.

**Result:**

The study included 127 cases, with 1 case withdrawal after enrollment due to a stroke of another determined cause, with 83 in the aspiration thrombectomy group and 43 cases in the stent retriever thrombectomy group. The change in NIHSS score from preoperative to immediate postoperative was 5 (1, 8) in the aspiration thrombectomy group and 1 (0, 4.5) in the stent retriever thrombectomy group. The change from preoperative to discharge was 8 (5, 12) in the aspiration thrombectomy group and 4 (0, 9) in the stent retriever thrombectomy group. The aspiration thrombectomy group exhibited significantly better prognosis rates and shorter time from successful femoral artery puncture to successful vessel recanalization. There were no significant differences between the two groups in terms of successful vessel recanalization rates and perioperative complications.

**Conclusion:**

As a first-line approach for anterior circulation large vessel cardiogenic cerebral embolism and cryptogenic stroke, aspiration thrombectomy leads to better improvement in neurological functional deficits and prognosis rates compared to stent retriever thrombectomy.

## Introduction

Cardiogenic cerebral embolism is a type of ischemic stroke caused by a cardiogenic embolus (or emboli) dislodged and embolized into the corresponding cerebral artery (1). Compared to other types of stroke, cardiogenic cerebral embolism progresses more rapidly and is accompanied by more severe complications and higher rates of adverse outcomes (1, 2). Ischemic stroke of undetermined etiology, often called cryptogenic stroke (3), shows strong overlap in both histopathologic thrombus characteristics and interventional and clinical outcome parameters with cardioembolic (4). HERME meta-analysis (5) demonstrated that patients who received endovascular therapy had better neurological outcomes than those who received medical treatment alone. Endovascular therapy currently includes two main techniques: aspiration thrombectomy and stent retriever thrombectomy, which can also be used in combination if necessary. However, it is unclear which technique should be selected as the first-line approach for endovascular therapy treatment. The ASTER and COMPASS (6, 7) studies showed no significant difference between these two techniques in terms of successful recanalization rates. Even in the ASTER2 and Penumbra 3-D (8, 9) studies, first-line aspiration was not superior to first-line stent retriever in achieving successful revascularization at the end of the endovascular procedure. American guidelines suggest that the use of standalone aspiration thrombectomy or combination with aspiration thrombectomy is reasonable, but stent retriever thrombectomy is still the preferred mechanical thrombectomy strategy (10). However, aspiration thrombectomy can reduce operation time and has the advantages of wide indications, simple operation, and cost-effectiveness. With the development of thrombectomy techniques and consumables in recent years, the conditions for aspiration thrombectomy are more mature, and the choice of which technique to use as the first-line approach remains controversial (11, 12). Based on the background outlined above, this study aims to compare the clinical efficacy and safety of aspiration thrombectomy and stent retriever thrombectomy as a first-line approach for cardiogenic cerebral embolism in large vessels of the anterior to provide guidance for the clinical selection of thrombectomy techniques.

## Materials and methods

### Research subjects

We conducted a retrospective observational study on patients diagnosed with cardiogenic cerebral embolism and cryptogenic stroke in large vessels of the anterior and treated with mechanical thrombectomy at our hospital from January 2020 to March 2023. Patients were divided into two groups based on the first-line approach they received: aspiration thrombectomy group and stent retriever thrombectomy group. Only when the first-line approach is unable to complete the vessel recanalization is another thrombectomy method used. All patients received equal perioperative care and routine treatment for stroke.

The study was approved by the ethics review committee of Guangzhou Panyu Central Hospital, with the ethics approval number PYRC-2023-077.

### Research methodology

After arriving at the hospital for the first time, all suspected stroke patients were given a detailed medical history inquiry and adequate communication and were evaluated using the National Institutes of Health Stroke Scale (NIHSS). Cranial imaging examinations were performed, including laboratory tests of blood biochemistry and coagulation function and CT + CTA of the cranial and neck or head MRI. If the conditions were met (13), the patients were treated with intravenous thrombolysis. All patients who had received thrombectomy were further evaluated by the surgical team to confirm surgical indications and exclude related contraindications to assess whether the patients would benefit from the thrombectomy procedure (14, 15). During the entire treatment process, all patients received extensive stroke-related examinations, such as CT + CTA of the cranial and neck, 24 h dynamic electrocardiography, echocardiography, laboratory tests, and daily NIHSS score assessments until discharge (16). Based on the results of the etiological screening, the patients were classified according to the Trial of Org 10,172 in Acute Stroke Treatment (TOAST) Classification (1). Perioperative antithrombotic medication was used based on the results of cranial imaging and etiological screening. In the angiography, tortuosity of the extracranial and cavernous internal carotid artery (ICA) was classified according to Junpei Koge’s study (17), and the location of occlusion was recorded.

**Figure 1:**
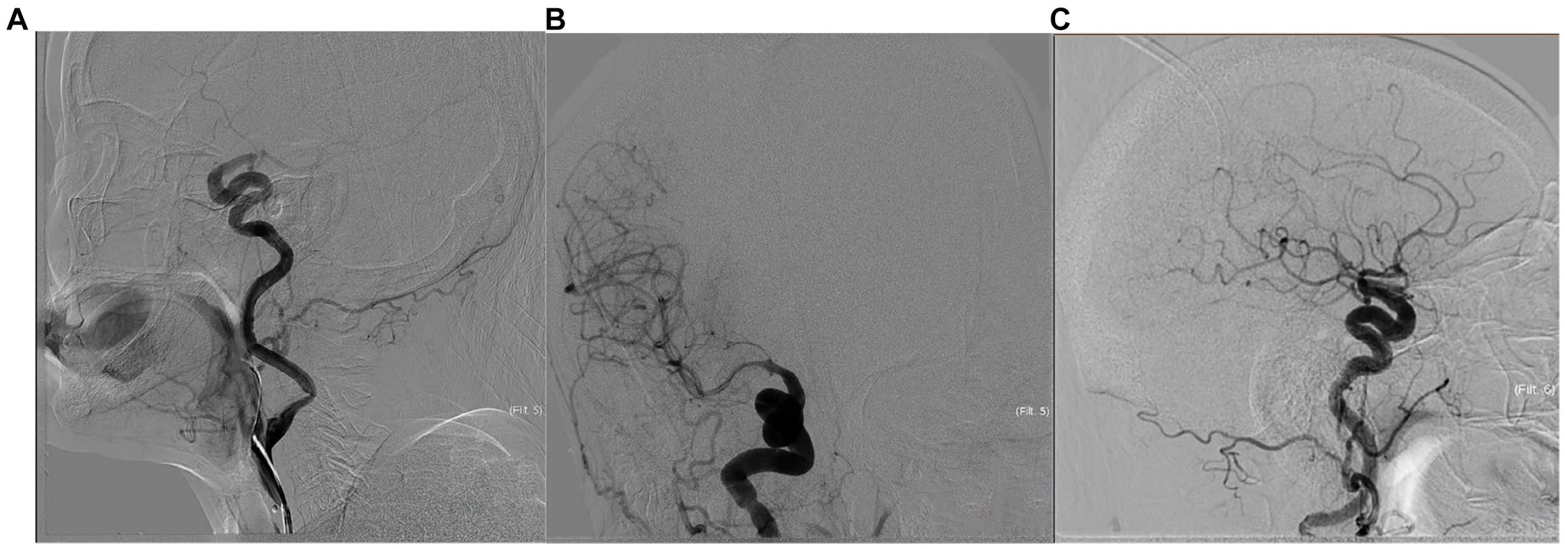
The patient is a 74-year-old woman who presented with right ICA occlusion, a preoperative NIHSS score of 17, with a history of atrial fibrillation suggestive of cardiogenic cerebral embolism. Emergent aspiration thrombectomy was performed as a first-line approach. The pre-treatment angiogram **(A)** showed right C7 segment occlusion. Tortuosity of extracranial ICA was kinked, and type III of cavernous ICA. The post-treatment angiogram after the first time aspiration thrombectomy **(B)** demonstrated reperfusion in the cervical and middle cerebral arteries but no visualization of the anterior cerebral artery. The post-treatment angiogram after the second time aspiration thrombectomy **(C)** showed full perfusion of the right anterior circulation with mTICI grade 3. The ADAPT technique was used in this procedure. The time from successful arterial puncture to reperfusion was 25 min. The patient’s NIHSS score improved to 13 immediately after the procedure, and further decreased to 1 at discharge.

**Figure 2:**
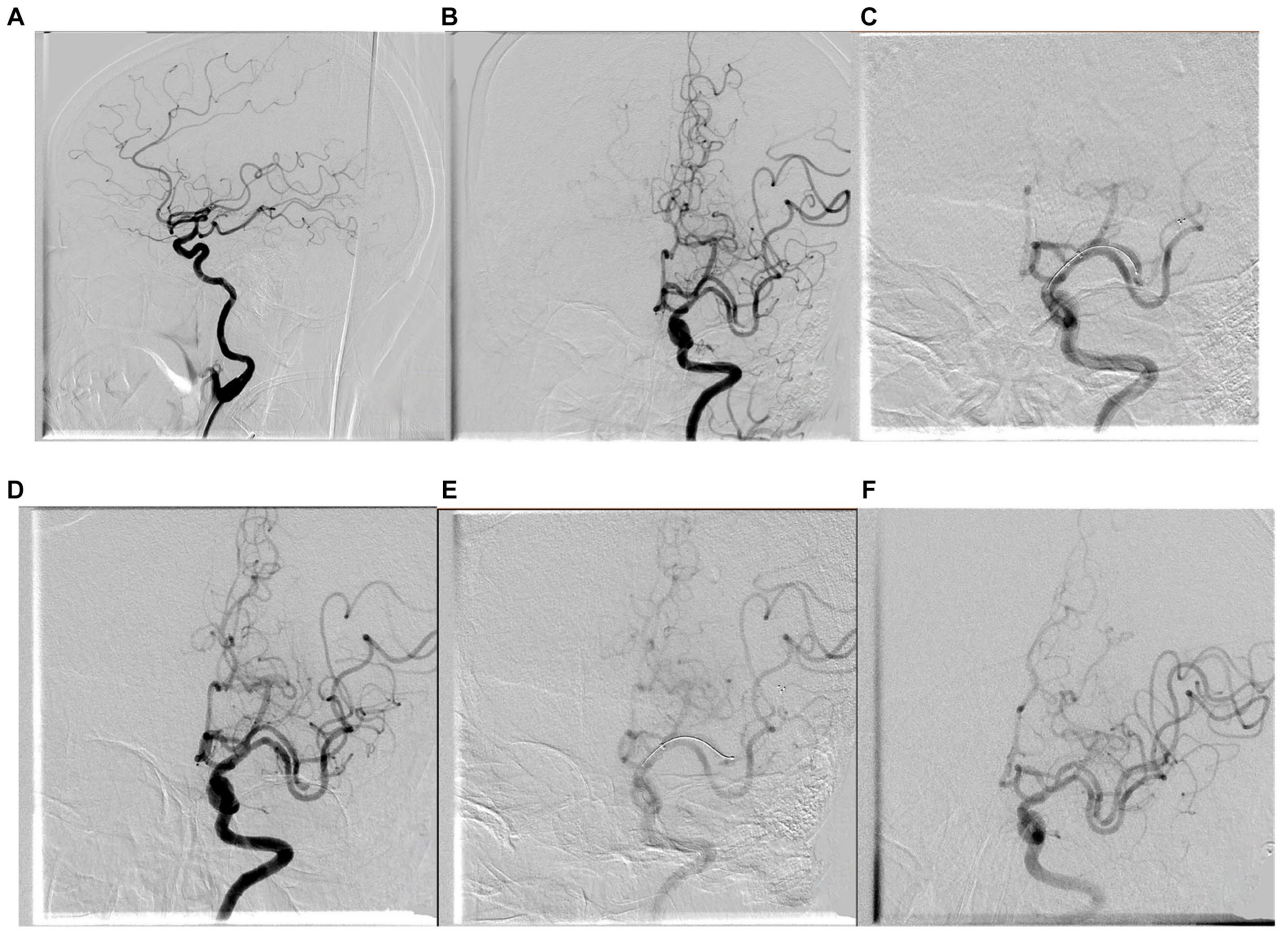
The patient is a 65-year-old woman who presented with left middle cerebral artery occlusion, a preoperative NIHSS score of 19, with a history of atrial fibrillation, suggestive of cardiogenic cerebral embolism. Emergent stent retriever thrombectomy was performed as a first-line approach. The pre-treatment angiogram **(A,B)** showed left M2 segment occlusion. Tortuosity of extracranial ICA was tortuous and type III of cavernous ICA. The first time treatment and post-treatment angiogram of stent retriever thrombectomy are shown in **(C,D)** and the second time shown in **(E,F)**. The Solumbra technique was used in this procedure. The embolus was broken and advanced, and there were occlusions in the M3 and M4 segments, and perfusion of the left middle cerebral artery returned to mTICI grade 2b. The time from successful arterial puncture to reperfusion was 54 min. The patient’s NIHSS score improved to 18 immediately after the procedure and further decreased to 17 at discharge.

The endovascular treatment procedure was as follows. The same coaxial was used in both aspiration and stent retriever thrombectomy, consisting of microguide wires (Traxcess, Synchro, Lunderquist), guiding catheter (Vista Brite Tip, Mach1, Envoy, Chaperon, Penumbra), distal access catheter (Sofia, Tethys), and sometimes included microcatheter (Rebar, Echelon, Headway, XT-27). The aspiration thrombectomy group used the ADAPT (11) technique, while the Solumbra (18, 19) or SWIM (20) technique was used in the stent retriever thrombectomy group. The distinction between the two groups lies in whether stent (Neurohawk, Solitaire AB, Syphonet) retriever thrombectomy was used as the first-line approach. It was up to the operator’s discretion whether to switch to another thrombectomy technique or other remedial measures. After the thrombectomy was completed, angiography was performed to clarify the presence of a thrombus, and dynaCT was performed to identify any hemorrhagic transformation (Figures 1, 2).

Inclusion criteria were as follows: (1) confirmation of acute ischemic stroke by imaging indicating anterior circulation large vessels occlusion; (2) preoperative Alberta Stroke Program Early CT (ASPECT) score ≥ 6 and cranial CT excluded intracranial hemorrhage; (3) the patients receive endovascular treatment within 6 h of onset or between 6 and 24 h after onset, following rigorous imaging screening; (4) signed informed consent from patients or their legal representatives; (5) diagnosis of cardiogenic cerebral embolism or cryptogenic stroke based on intraoperative imaging and postoperative echocardiography, transesophageal echocardiography, 24 h dynamic electrocardiogram, and transcranial Doppler bubble test; and (6) and preoperative mRS score ≤ 2. Exclusion criteria were as follows: (1) severe active bleeding or known significant bleeding tendency; (2) serious insufficiency of heart, liver, kidney, and other organs; (3) preoperative ASPECT score < 6 or preoperative imaging indicating intracranial hemorrhage; (4) expected survival was less than 90 days based on the patient’s medical data and examination results; and (5) preoperative mRS score > 2.

### Outcome measure

The primary outcome measure is the difference in immediate postoperative NIHSS scores and NIHSS scores at discharge compared to preoperative NIHSS scores between the two groups. The secondary outcome measures included the rate of good prognosis (mRS score ≤ 2) at discharge and 90 days, successful recanalization rate after thrombectomy (mTICI score ≥ 2b), time from successful femoral artery puncture to recanalization, and perioperative complication rate. Symptomatic intracerebral hemorrhage was evaluated by ECASS II criteria (21).

### Statistical analysis

SPSS20.0 software was used for statistical analysis. For continuous variables, the normality hypothesis test and the Shapiro–Wilk test were used. If both groups followed a normal distribution, descriptive statistics were presented as mean ± standard deviation ( $\bar{X}$ ± s), and an independent sample *t*-test was used for mean comparisons. Before the *t*-test, the homogeneity of variances was tested using the *F*-test. If the variances were homogeneous, the *t*-test was applied. If the variances were not homogeneous, the Welch’s *t*-test was used. If some or all continuous variables did not follow a normal distribution, the Mann–Whitney U-test was used, and the median (Q1, Q3) was reported. For ordinal categorical variables, the Pearson’s chi-squared test was used. For small-sample non-ordinal categorical variables between two groups, the Mann–Whitney U-test was used, and the results were presented as counts (percentages) [*n* (%)]. *p* < 0.05 was considered as the standard of statistical significance.

### Data availability

Any data not published within the article will be shared by the qualified investigators on reasonable request.

## Results

### Baseline data

A total of 127 cases were enrolled in this study, with 1 case withdrawal after enrollment due to artery-to-artery embolism. Among them, 8 cases with relapsed embolism received a total of two thrombectomy treatments. Among the 83 cases that received aspiration thrombectomy as the first-line treatment, 9 cases that received general anesthesia could not complete the immediate postoperative NIHSS assessment, and 12 cases had missing data on the time from stroke onset to successful femoral artery puncture due to wake-up stroke. The 43 cases were treated with stent retriever thrombectomy as the first-line treatment, with 2 cases unable to complete immediate postoperative NIHSS assessment due to general anesthesia and 6 cases missing data on the time from stroke onset to successful femoral artery puncture due to wake-up stroke. There were no statistically significant differences (*p* > 0.05) in baseline data projects of age, gender, medical history, current stroke event, etiology of embolism, treatment procedures, or tortuosity of ICA, indicating comparability. However, there were statistically significant differences in the location of occlusion (*p* < 0.05). Specific clinical baseline data are presented in Table 1.

**Table 1:** Comparison of baseline clinical data between the two groups.

Projects	Aspiration thrombectomy group (*n* = 83)	stent retriever thrombectomy group (*n* = 43)	*χ*^2^/ *Z*	*p*
Male [*n* (%)]	36 (43.4)	20 (46.5)	0.113	0.737
Age [years, *M* (*Q*_1_*, Q*_3_)]	67 (58, 74)	71 (64, 77)	−1.936	0.053
Medical history [*n* (%)]				
Hypertension [*n* (%)]	51 (61.5)	26 (60.5)	0.011	0.915
Diabetes mellitus [*n* (%)]	10 (12.0)	7 (15.9)	0.434	0.510
History of stroke [*n* (%)]	17 (20.5)	10 (23.3)	0.129	0.719
Current stroke events				
Preoperative NIHSS [score, *M* (*Q*_1_*, Q*_3_)]	13 (10, 19)	14 (9, 18)	−0.003	0.998
Pre-onset mRS [score, *M* (*Q*_1_*, Q*_3_)]	0 (0, 0)	0 (0, 0)	−0.545	0.586
ASPECTS [score, *M* (*Q*_1_*, Q*_3_)]	9 (7, 9)	8 (7, 9)	−1.254	0.210
Time from onset to successful femoral artery puncture [min, *M* (*Q*_1_*, Q*_3_)]	210 (140, 260)^a^	217.5 (159, 343.5)^b^	−1.195	0.232
Twice embolism [*n* (%)]	5 (6.0)	3 (7.0)	0.043	0.835
Etiology				
Cardioembolic [*n* (%)]	60 (72.3)	32 (74.4)	0.065	0.798
Cryptogenic etiology [*n* (%)]	23 (27.7)	8 (25.6)		
Treatment procedures				
Intravenous thrombolysis with Alteplase [*n* (%)]	28 (33.7)	18 (41.9)	0.807	0.369
General anesthesia [*n* (%)]	11 (13.3)	4 (9.3)	0.422	0.516
Balloon dilation [*n* (%)]	1 (1.2)	1 (2.3)	0.228	0.633
Stent angioplasty [*n* (%)]	2 (2.4)	1 (2.3)	0.001	0.977
Intraarterial perfusion of tirofiban [*n* (%)]	3 (3.6)	5 (11.6)	3.059	0.080
Location of occlusion				
C1 [*n* (%)]	5 (6.0)	0 (0)	2.697	0.101
C2 [*n* (%)]	6 (7.2)	0 (0)	3.305	0.069
C3 [*n* (%)]	5 (6.0)	0 (0)	2.697	0.101
C4 [*n* (%)]	9 (10.8)	0 (0)	5.021	0.025
C5 [*n* (%)]	13 (15.7)	0 (0)	7.510	0.006
C6 [*n* (%)]	18 (21.7)	1 (2.3)	8.292	0.004
C7 [*n* (%)]	24 (28.9)	7 (16.3)	2.438	0.118
M1 [*n* (%)]	61 (73.5)	24 (55.8)	4.033	0.045
M2 [*n* (%)]	25 (30.1)	25 (58.1)	9.291	0.002
M3 [*n* (%)]	5 (6.0)	2 (4.7)	0.102	0.750
A1 [*n* (%)]	1 (1.2)	0 (0)	0.522	0.470
A2 [*n* (%)]	1 (1.2)	2 (4.7)	1.447	0.229
A3 [*n* (%)]	0 (0)	0 (0)	–	–
Extracranial ICA tortuosity				
Straight [*n* (%)]	19 (22.9)	7 (16.3)	−0.696	0.487
Tortuous [*n* (%)]	58 (69.9)	33 (76.7)		
Coiled [*n* (%)]	4 (4.8)	2 (4.7)		
Kinked [*n* (%)]	2 (2.4)	1 (2.3)		
Cavernous ICA tortuosity				
Type I [*n* (%)]	40 (48.2)	19 (44.2)	−0.749	0.454
Type II [*n* (%)]	32 (38.6)	16 (37.2)		
Type III [*n* (%)]	11 (13.3)	4 (9.3)		
Type IV [*n* (%)]	0 (0)	4 (9.3)		

a *n* = 71.

b *n* = 38.

NIHSS, National Institutes of Health Stroke Scale; mRS, Modified Rankin Scale; ASPECT, Alberta Stroke Program Early CT Score; ICA, internal carotid artery.

### Primary and secondary outcomes

For the primary outcome, the change in NIHSS scores from preoperative to immediate postoperative was 5 (1, 8) in the aspiration thrombectomy group and 1 (0, 5) in the stent retriever thrombectomy group, with a statistically significant difference (*Z* = −3.597, *p* = 0.000). The change in NIHSS scores from preoperative to discharge was 8 (5, 12) in the aspiration thrombectomy group and 4 (0, 9) in the stent retriever thrombectomy group, with a statistically significant difference (*Z* = −2.327, *p* = 0.020).

For the secondary outcome, a good prognosis was defined as mRS score ≤ 2. The rate of good prognosis at discharge in the aspiration thrombectomy group was 46 cases (55.4%), while it was 14 cases (31.8%) in the stent retriever thrombectomy group, showing a statistically significant difference (χ^2^ = 7.358, *p* = 0.025). The rate of good prognosis at 90 days in the aspiration thrombectomy group was 49 cases (63.6%), while it was 17 cases (41.5%) in the stent retriever thrombectomy group, showing a statistically significant difference (χ^2^ = 5.336, *p* = 0.021).

Regarding the mTICI score ≥ 2b, indicating successful vessel recanalization, there were 80 cases (96.4%) in the aspiration thrombectomy group and 40 cases (93.0%) in the stent retriever thrombectomy group, with no statistically significant difference (χ^2^ = 0.706, *p* = 0.401).

Regarding the time from successful femoral artery puncture to successful vessel recanalization, the aspiration thrombectomy group had a median time of 22 (16, 36) min, while the stent retriever thrombectomy group had a median time of 39 (28, 54) min, showing a statistically significant difference (*Z* = −4.213, *p* = 0.000).

In terms of perioperative complication rates, the aspiration thrombectomy group reported 20 cases of extensive cerebral infarction, 1 case of Symptomatic intracerebral hemorrhage, 6 cases of coma, and 3 cases of death. The stent retriever thrombectomy group reported 13 cases of extensive cerebral infarction, 2 cases of symptomatic intracerebral hemorrhage, 2 cases of coma, and 0 cases of death. There were no statistically significant differences in these complications between the two groups (*p* > 0.05). Specific clinical efficacy indicator data are presented in Table 2.

**Table 2:** Comparison of clinical efficacy indexes between the two groups.

Projects	Aspiration thrombectomy group (*n* = 83)	Stent retriever thrombectomy group (*n* = 43)	*χ*^2^/ *Z*	*p*
NIHSS score Change from preoperative to immediate postoperative [score, *M* (*Q*1, *Q*3)]	5 (1, 8)^a^	1 (0, 4.5)^b^	−3.597	0.000
NIHSS score Change from preoperative to discharge [score, *M* (*Q*1, *Q*3)]	8 (5, 12)	4 (0, 9)	−2.327	0.020
Good prognosis at discharge [*n* (%)]	46 (55.4)	14 (31.8)	7.358	0.025
Good prognosis at 90d [*n* (%)]	49 (59.0)^c^	17 (40.9)^d^	5.336	0.021
Successful vessel revascularization rate [*n* (%)]	80 (96.4)	40 (93)	0.706	0.401
Time from successful femoral artery puncture to successful vessel revascularization [min, *M* (*Q*1, *Q*3)]	22 (16, 36)	39 (28, 54)	−4.213	0.000
Perioperative complications				
Extensive cerebral infarction [*n* (%)]	20 (24.1)	13 (30.2)	0.552	0.458
Symptomatic intracerebral hemorrhage [*n* (%)]	1 (1.2)	2 (4.7)	1.447	0.229
Coma [*n* (%)]	6 (7.2)	2 (4.7)	0.317	0.574
Death [*n* (%)]	3 (3.6)	0 (0)	1.592	0.207

a *n* = 74.

b *n* = 41.

c *n* = 77.

d *n* = 41.

NIHSS, National Institutes of Health Stroke Scale.

## Discussion

This is a single-center retrospective observational study that included 127 patients diagnosed with anterior circulation cardiogenic cerebral embolism and treated with endovascular therapy. Based on the study outcomes, it was observed that using aspiration thrombectomy as the first-line approach could improve patients’ neurological function and result in a better rate of good prognosis compared to stent retriever thrombectomy.

The NIHSS score is a reliable tool for assessing neurological deficits in patients with anterior circulation ischemic stroke and is considered the primary indicator that best reflects treatment outcomes. Even the mTICI score after thrombectomy can effectively reflect the immediate treatment effect and is closely related to the patient’s neurological function outcome. However, there might not be a significant difference in the reperfusion rates between the two procedures, as observed in our study, which is consistent with some previous research (6–9). However, aspiration thrombectomy significantly shortens the time to reperfusion. “Time is brain,” aspiration thrombectomy is a simple, fast, and effective procedure that can reduce the time to reperfusion without increasing perioperative complications, which may lead to better improvement in neurological deficits and patient prognosis (11, 19, 22). Although some studies have suggested that aspiration thrombectomy may require more remedial measures, which could be associated with worse research outcomes (23), it might be due to atherosclerosis-caused arterial stenosis (19), while only cardiogenic cerebral embolism and cryptogenic stroke were included in our study.

In addition, repeated mechanical shear forces are inevitably applied to the vessel wall, leading to potential injuries (24). These endothelium injuries can stimulate the proliferation and migration of vascular smooth muscle cells, resulting in abnormal intimal hyperplasia and restricted blood flow. Clinical studies have indicated that repeated stent retriever thrombectomy is associated with a higher risk of reocclusion, vasospasm, and vascular dissection (25). Compared to stent retriever thrombectomy, aspiration thrombectomy with simpler procedure and shorter retrieval time reduces the risk of intravascular injury, leading to better improvement in neurological deficits and patient prognosis.

In addition, the structure for thrombi of cardiogenic cerebral embolism is different from non-cardiogenic. Cardioembolic emboli are the origin of the heart cavity, valves, or other places in the heart. When the blood is slow or stagnant and the fibrin of the vascular wall is exposed, the coagulation pathway will be activated, potentiating the formation of a red thrombus. Atrial fibrillation, myxoma, patent foramen ovale, and other prone to produce red thrombosis (3), so-called cryptogenic strokes, are cardioembolic (2, 3). Meanwhile, a white thrombus is caused by platelet activation and aggregation caused by the rupture of arterial plaque (26). Compared to emboli from large-artery atherosclerosis, and undetermined etiology stroke, cardioembolic emboli have a higher content of red blood cells and organizational degree of these thrombi, with larger volumes, leading to poorer clinical manifestations and prognosis, requiring a higher number of retraction maneuvers (2, 4). In the Chinese thrombectomy guidelines, emboli with large volumes are recommended to be treated with a large bore catheter for aspiration thrombectomy.

Furthermore, advancements in materials will further propel the development of aspiration thrombectomy techniques. Innovations in large bore catheters, represented by the Sofia catheter, have been used frequently (11, 19, 22). In addition, adhering to the principle of “Bigger is better,” Millipede 088 aspiration catheters have shown a good development prospect when it comes to aspiration catheters in appropriately sized vessels (27). Moreover, the development of aspiration catheters such as 3MAX, with increased suction force, improved flexibility, and better resistance to bending, enables access to more distal and smaller vessels such as the pericallosal artery and the callosomarginal artery with thinner vascular walls (28). These advancements will lead to even better outcomes in terms of thrombus removal, making aspiration thrombectomy an even more attractive and promising treatment option for cardiogenic cerebral embolism and cryptogenic stroke.

## Limitation

Although this study produced significant results, these findings may be based on biases. The study lacked randomization in assignment, and the choice of thrombectomy technique depended on the operator’s preference. For instance, aspiration thrombectomy was preferred for ICA and M1 segments, while stent retriever thrombectomy was more commonly used for the M2 segment. These factors inevitably introduce bias into the study. Prospective double-blind trials are still ongoing. Under this premise, we concluded that aspiration thrombectomy has better efficacy, and it did not increase the probability of requiring remedial measures.

A recent study found that intravenous thrombolysis could influence certain efficacy outcomes of stent retrievers or aspiration thrombectomy (29). However, due to a limited number of cases, we could not conduct a dedicated study to eliminate this potential confounding factor, although there may not be a significant difference in intravenous thrombolysis between the two groups in baseline data.

## Conclusion

For patients with anterior circulation large vessel cardiogenic cerebral embolism and cryptogenic stroke, aspiration thrombectomy as the first-line approach provides better improvement in neurological deficits and prognosis compared to stent retriever thrombectomy. It also results in a shorter time to achieve successful revascularization, with no significant differences observed between the two groups in terms of revascularization rates and perioperative complications.

## Data availability statement

The raw data supporting the conclusions of this article will be made available by the authors, without undue reservation.

## Ethics statement

The studies involving humans were approved by Medical Ethics Review of Guangzhou Panyu Central Hospital. Ethics approval number PYRC-2023-077. The studies were conducted in accordance with the local legislation and institutional requirements. Written informed consent for participation was not required from the participants or the participants’ legal guardians/next of kin because retrospective observational study.

## Author contributions

PZ: Conceptualization, Funding acquisition, Writing – review & editing. CJ: Conceptualization, Data curation, Formal analysis, Writing – original draft. CW: Conceptualization, Methodology, Writing – review & editing. ZD: Conceptualization, Methodology, Writing – review & editing. MM: Conceptualization, Writing – review & editing. LQ: Conceptualization, Writing – review & editing. ZZ: Funding acquisition, Writing – review & editing. HW: Data curation, Writing – review & editing. YB: Conceptualization, Writing – review & editing. CM: Conceptualization, Data curation, Writing – review & editing.
